# Spontaneously opening GABA_A_ receptors play a significant role in neuronal signal filtering and integration

**DOI:** 10.1038/s41419-018-0856-7

**Published:** 2018-07-24

**Authors:** Nathanael O’Neill, Sergiy Sylantyev

**Affiliations:** 1 CCBS, University of Edinburgh, 49 Little France Crescent, Edinburgh, EH16 4SB UK; 2 DCEE, Institute of Neurology, University College London, QSH, Queen Square, London, WC1N 3BG UK

## Abstract

Continuous (tonic) charge transfer through ionotropic receptors of *γ*-aminobutyric acid (GABA_A_Rs) is an important mechanism of inhibitory signalling in the brain. The conventional view has been that tonic GABA-ergic inhibitory currents are mediated by low concentrations of ambient GABA. Recently, however, it was shown that the GABA-independent, spontaneously opening GABA_A_Rs (s-GABA_A_Rs), may contribute significantly to the tonic GABA_A_R current. One of the common approaches to temporal lobe epilepsy (TLE) therapy is an increase of GABA concentration in the cerebrospinal fluid to augment tonic current through GABA_A_Rs. Such an increase, however, generates multiple side effects, which impose significant limitations on the use of correspondent drugs. In contrast, activation/deactivation of s-GABA_A_Rs in a GABA-independent manner may provide a mechanism of regulation of tonic conductance without modification of extracellular GABA concentration, thus avoiding connected side effects. Although s-GABA_A_Rs have been detected in our earlier work, it is unclear whether they modulate neural signalling, or, due to their independence from the neurotransmitter, they provide just a stable background effect without much impact on neural crosstalk dynamics. Here, we focused on the causal relationship between s-GABA_A_R activity and signal integration in the rat’s dentate gyrus granule cells to find that s-GABA_A_Rs play an important role in neural signal transduction. s-GABA_A_Rs shape the dynamics of phasic inhibitory responses, regulate the action potential generation machinery and control the coincidence detection window pertinent to excitatory input summation. Our results demonstrate that tonic inhibition delivered by s-GABA_A_Rs contributes to the key mechanisms that ensure implementation of neural signal filtering and integration, in a GABA-independent manner. This makes s-GABA_A_R a new and important actor in the regulation of long-term neural plasticity and a perspective target for TLE therapy.

## Introduction

The classical trigger of current flow through ionotropic *γ*-aminobutyric acid receptors (GABA_A_Rs) is the binding of GABA released from the presynaptic terminal, which induces short, concerted openings of GABA_A_Rs to mediate phasic inhibition. Another mechanism of inhibitory signalling is charge transfer via tonically (continuously) active GABA_A_Rs. Studies on tonic inhibitory conductance through GABA_A_Rs attract substantial attention due to their profound influence on neural excitability, synaptic plasticity, neurogenesis, and network oscillations^1–4^. The relatively understudied form of tonic inhibition is mediated by constitutively active GABA_A_Rs, i.e., receptors that open spontaneously in the absence of GABA^2^.

In our recent study on dentate gyrus granule cells (DGCs), we demonstrated that when the extracellular concentration of GABA was matched to the ambient levels measured in vivo, the vast majority (~90%) of tonic inhibition was delivered by spontaneously opening GABA_A_Rs (s-GABA_A_Rs)^5^. s-GABA_A_Rs do not require the binding of GABA to enter an active state and, therefore, are resistant to block by competitive antagonists with negligible negative efficacy (SR-95531, SR). However, s-GABA_A_Rs can be inhibited by bicuculline (BIC), which exhibits negative efficacy (inverse agonist)^6^ and open-channel blockers, e.g., picrotoxin (PTX) and pentylenetetrazole (PTZ). In the aforementioned study, we showed that SR can reverse the effects of BIC. This important finding demonstrates that the lack of effect of SR-95531 is not simply due to a failure to bind to GABA_A_Rs, but due to the lack of negative efficacy^6^.

The hippocampus is a brain area especially prone to epilepsy, and DGCs are commonly affected in a course of temporal lobe epilepsy (TLE) development^7,8^. It was shown that phasic GABA-ergic inhibition is reduced in TLE, whereas the tonic GABA-ergic conductance remains intact^9,10^. This makes tonic GABA-ergic current an attractive target for anti-TLE treatment. The obvious treatment approach is to increase the concentration of extracellular GABA, which activates tonically active GABA_A_Rs. However, this approach was repeatedly found to be ineffective^11,12^ or even leading to epileptogenesis^9,13^ due to various side effects. In contrast, modulation of s-GABA_A_Rs may offer an alternative approach for the treatment of TLE, by way of augmenting tonic inhibition without the need to change extracellular GABA concentration, thus avoiding the associated off-target effects.

To date, almost nothing is known about the functional impact of this GABA-independent conductance on neuron-firing characteristics and integration of synaptic inputs. In addition, failure to register s-GABA_A_Rs openings in outside-out patches^5^ raises a question whether s-GABA_A_Rs activation depends critically from some cytoplasmic factors.

In the present study, we aimed to establish what generic role(s) s-GABA_A_Rs play in regulating the synaptic circuits in the dentate gyrus and provide a new perspective target for TLE therapy. We hypothesized that s-GABA_A_Rs provide a persistent reduction in input resistance, which acts as a break on excitability and narrows the temporal window with which excitatory inputs can be summated to produce an action potential. The dentate gyrus acts as a regulatory “gate” into the hippocampus, serving to filter and separate inputs^14^. Henceforth, even subtle changes to excitability could have a marked effect on the long-term hippocampal plasticity^15^. Lastly, we also tried to clarify if s-GABA_A_Rs are capable of being gated by GABA, as the case in recombinant expression systems^16–18^.

## Results

### s-GABA_A_Rs deliver a major part of inhibitory conductance

As the first step of our study, we assessed whether s-GABA_A_Rs make a significant contribution to the overall inhibitory conductance in DGCs. To distinguish between GABA-dependent and GABA-independent effects of GABA_A_Rs, we used differences in the mechanism of action of SR and PTX. SR has been shown to be a competitive antagonist with no negative efficacy^6^ and thus abolishes GABA_A_R activity induced by GABA binding, i.e., acts on conventional (GABA-dependent) GABA_A_Rs; conversely, PTX binds inside the GABA_A_R channel and thus blocks all GABA_A_Rs that enter active state, i.e., acts on both conventional GABA_A_Rs and s-GABA_A_Rs. Therefore, here and in further experiments, we exploited SR and PTX to measure the activity of conventional GABA_A_Rs and s-GABA_A_Rs. Specifically, conventional GABA_A_R activity was assessed as the change in the given effect obtained under control vs. after application of SR, whereas s-GABA_A_R activity was measured as the change in the effect obtained after SR application vs. after subsequent application of SR + PTX.

**Fig. 1:**
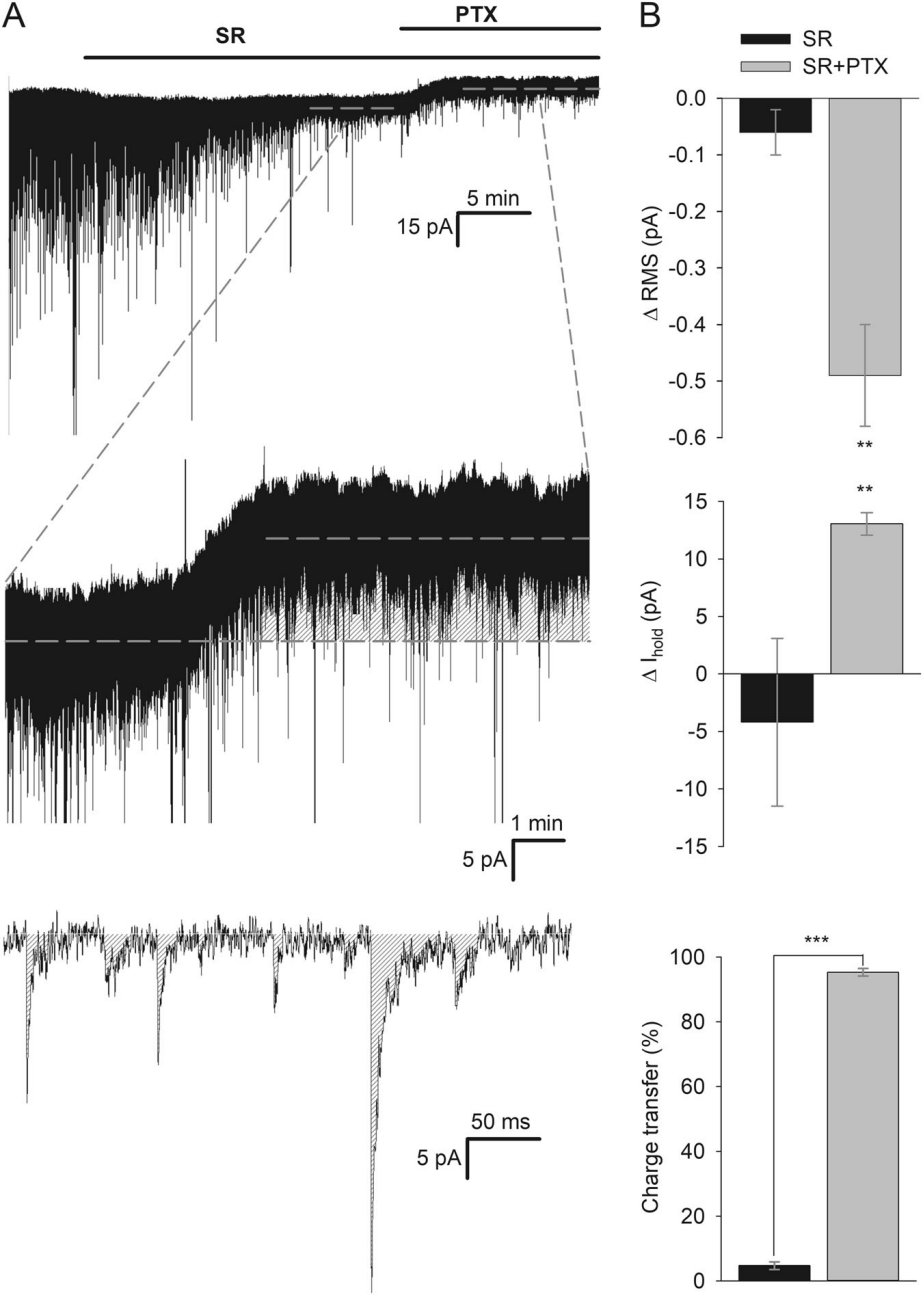
s-GABA_A_Rs control a major part of inhibitory charge transfer. **a** Example traces of whole-cell continuous recordings from DGCs. Top: Full experimental trace; application of SR (25 μM) blocks GABA-dependent phasic conductance, whereas subsequent application of SR + PTX (50 μM) blocks tonic conductance controlled by s-GABA_A_Rs. Horizontal dashed lines mark time intervals over which *I*_tonic_ was averaged for SR and SR + PTX. Medium: Expanded section of the recording demonstrates the effect of s-GABA_A_Rs blocked by PTX. Shaded area shows the amount of tonic current controlled by s-GABA_A_Rs. Bottom: Expanded 0.5-s recording interval before application of SR. Shaded area shows the amount of inhibitory current transferred with GABA-induced phasic events. **b** Statistical summary on effects of s-GABA_A_Rs and GABA-dependent effects. Top: SR and SR + PTX decrease RMS noise; bars represent change (ΔRMS) from RMS in control (for SR) and from RMS under SR (for SR + PTX), asterisks indicate significance of difference from zero; *n* = 6, Student’s paired *t* test. Medium: Shifts of *I*_hold_ induced by the application of SR and SR + PTX; Δ*I*_hold_ for SR and SR + PTX was calculated similarly to ΔRMS. Asterisks indicate significance of difference from zero; *n* = 6, Student’s paired *t* test. Bottom: Input into inhibitory charge transfer delivered by GABA-dependent GABA_A_Rs (phasic events suppressed by SR) and s-GABA_A_Rs (tonic response suppressed by SR + PTX). *n* = 6, Student’s paired *t* test. Colour codes apply for all three bar charts, **P < 0.01, ***P < 0.001

To quantify s-GABA_A_R contribution to inhibitory signalling, we performed continuous whole-cell recordings, registering changes in RMS noise (ΔRMS), holding current (Δ*I*_hold_) and inhibitory charge transfer (Fig. 1).

SR induced ΔRMS of −0.06 ± 0.04 pA when compared with control value of 3.01 ± 0.26 pA, whereas SR + PTX induced much larger RMS decrease when compared with SR alone: ΔRMS = −0.49 ± 0.09 pA (Fig. 1b). One-way ANOVA demonstrated significant influence of GABA_A_R ligands on RMS: F_(2,16)_ = 4.23, *P* = 0.033; Student–Newman–Keuls post hoc test (SNK): *P* < 0.05 for Control vs. SR + PTX and for SR vs. SR + PTX.

Similarly, SR induced only a nominal change of −74.9 ± 19.4 pA holding current observed in the control: Δ*I*_hold_ SR vs. control = −4.2 ± 7.3 pA; on the contrary, the addition of PTX induced much larger effect: Δ*I*_hold_ SR + PTX vs. SR = 13.05 ± 0.97 pA, Fig. 1b. One-way ANOVA again confirmed significance of GABA_A_R ligands effect: F_(2,16)_ = 4.74, *P* = 0.024; SNK: *P* < 0.05 for Control vs. SR + PTX and for SR vs. SR + PTX.

When we compared charge transfer through GABA-activated GABA_A_Rs (spontaneous post-synaptic currents, sIPSCs) suppressed by SR and charge transfer through s-GABA_A_Rs suppressed by SR + PTX, we found that s-GABA_A_Rs make a significant contribution to overall inhibitory charge transfer, far surpassing that delivered by sIPSCs: 95.3 ± 1.2% vs. 4.7 ± 1.2%, *P* < 0.0001, *n* = 6, Student’s paired *t* test (Fig. 1b).

### Single-channel properties of s-GABA_A_Rs differ from that of GABA-activated GABA_A_Rs

We next assessed whether s-GABA_A_Rs effects and GABA-triggered effects of GABA_A_Rs can be separated by their single-channel characteristics (conductance, opening frequency and average open-time).

**Fig. 2:**
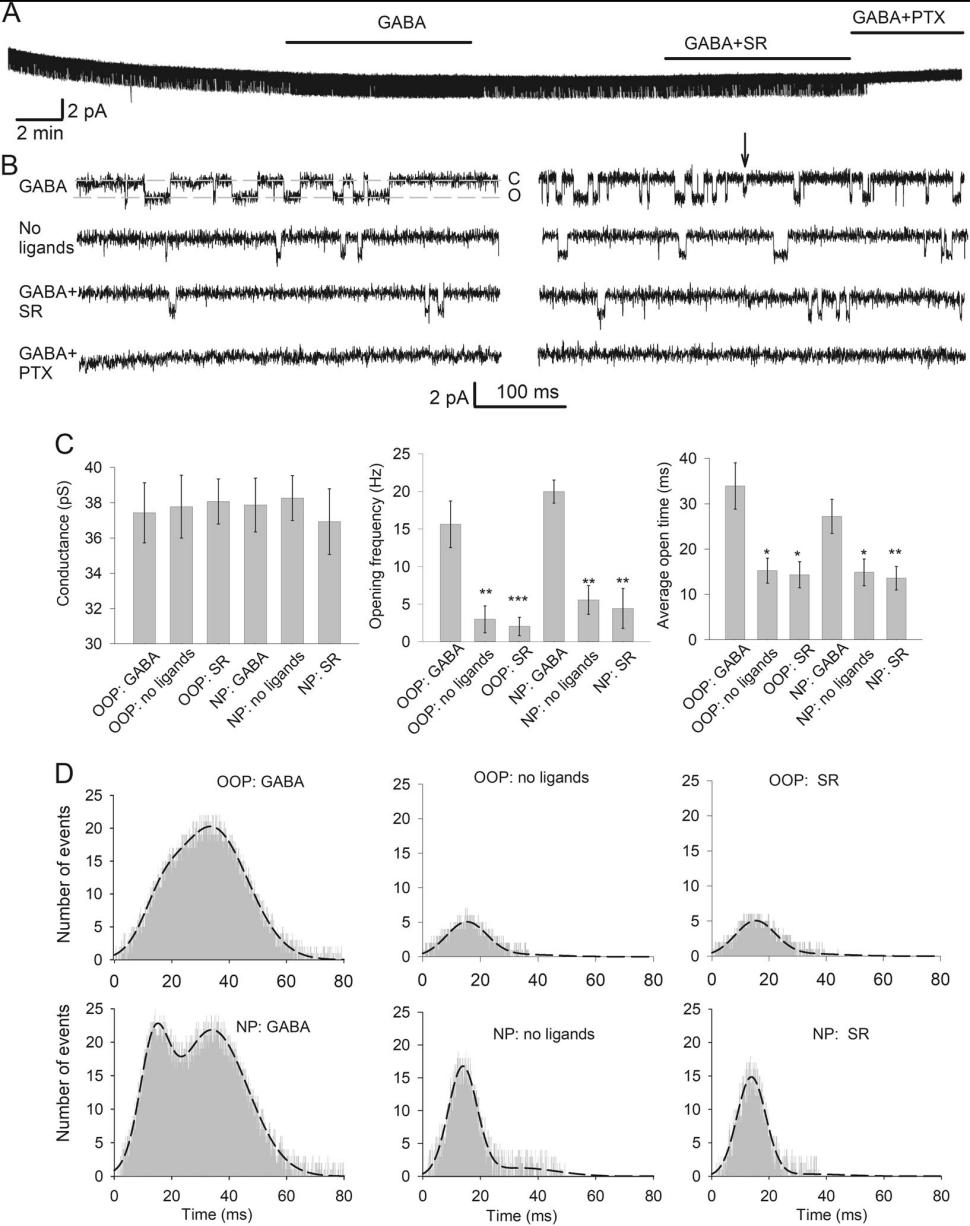
s-GABA_A_Rs can be distinguished from conventional GABA_A_Rs by opening frequency and average open-time. **a** Example trace of single-channel activity in the membrane patch with a sequence of GABAR ligand application. **b** Single-channel GABA_A_Rs openings in membrane patches. Left: outside-out patch. Right: nucleated patch, pulled from the same neuron. “C” and “O” denote closed and open states of the receptor, respectively. Arrow indicates low-conductance receptor opening. Traces from top to bottom: GABA (10 μM), perfusion solution without GABA_A_R ligands, GABA (10 μM) + SR (25 μM) and GABA (10 μM) + PTX (50 μM). Scale bars apply to all traces. **c** Statistical summary on single-channel parameters recorded in **b**. Asterisks show significance of difference from the value generated by GABA only at corresponding patch type. *P < 0.05, **P < 0.01, ***P < 0.001, *n* = 6, Student’s paired *t* test. **d** All-points open-time histograms for receptor openings recorded in **b**. Axes legends apply to all histograms. OOP: outside-out patch, NP: nucleated patch

GABA_A_R openings were recorded first from the outside-out patch (OOP) and then from the nucleated patch (NP), pulled from the same neuron (Fig. 2). Application of 10 μM GABA generated single-channel activity in both patch types (Fig. 2а, b). Conversely, in the absence of exogenous GABA, the majority of cells (five out of six) did not exhibit any spontaneous openings in the OOP configuration. However, with low-impedance pipettes (~3 MΩ), we could detect GABA-independent openings in OOPs.

In both OOPs and NPs, the absence of GABA and the application of SR (25 μM) left intact the part of the single-channel activity, whereas PTX (50 μM) blocked it in full (Fig. 2b).

Single-channel conductance was indistinguishable in OOPs and NPs under all experimental conditions (varying from 38.3 ± 1.3 to 36.9 ± 1.9 pS, Fig. 2c), which does not allow distinguishing s-GABA_A_Rs from GABA-activated GABA_A_Rs. Instead, s-GABA_A_Rs can be isolated pharmacologically by withdrawal of GABA and/or application of SR, which cause a significant decrease in the receptor-opening frequency and average open-time. In OOPs, the opening frequency decreased from 15.6 ± 3.1 Hz obtained with GABA in the perfusion solution to 2.98 ± 1.78 Hz without GABA (*P* = 0.0084, *n* = 6, Student’s paired *t* test), and to 2.02 ± 1.23 Hz under SR (*P* = 0.0092, *n* = 6, Student’s paired *t* test). In NPs, the opening frequency decreased from 19.98 ± 1.84 Hz generated by GABA to 5.54 ± 1.92 Hz and 4.42 ± 2.64 Hz when GABA was withdrawn and SR was added (*P* = 0.0089 and *P* = 0.0071, respectively, *n* = 6 in both cases, Student’s paired *t* test). The average open-time was also significantly decreased by pharmacological interventions: in OOPs, from 33.93 ± 5.1 ms under GABA to 16.37 ± 2.78 ms with no GABA, and 14.35 ± 2.85 ms under SR (*P* = 0.038 and *P* = 0.029, respectively, *n* = 6, Student’s paired *t* test). In NPs, the average open-time decreased from 27.24 ± 3.76 ms under GABA to 15.92 ± 2.96 ms with no GABA, and 14.27 ± 2.6 ms with SR (*P* = 0.041 and *P* = 0.037, respectively, *n* = 6 for both cases, Student’s paired *t* test; Fig. 2c).

**Fig. 3:**
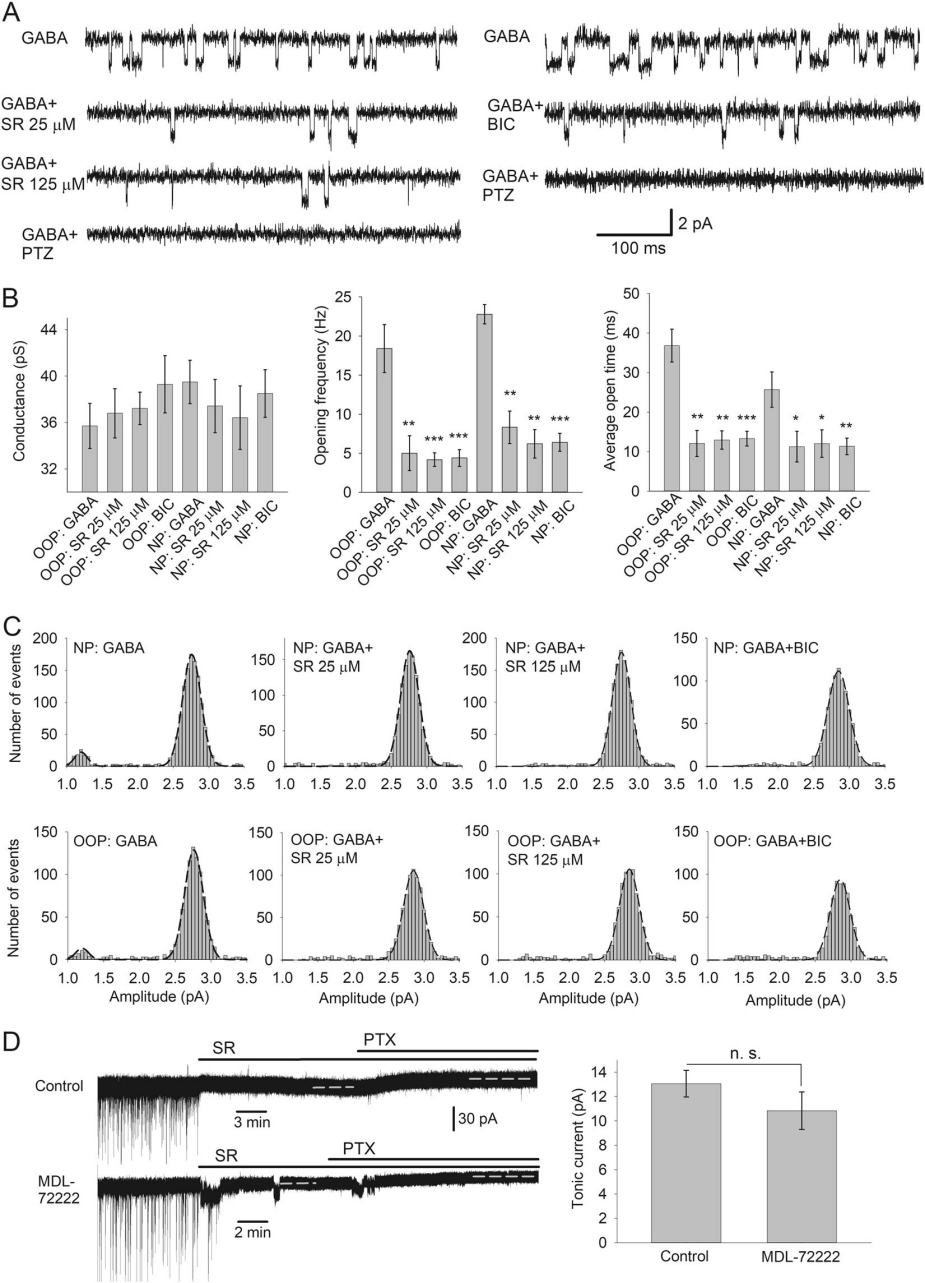
No significant side effects are generated by GABA_A_R ligands. **a** Example traces of single-channel GABA_A_Rs openings in membrane patches. Left: Effects of incrementing concentrations of SR and PTZ (100 μM). Right: Effects of BIC (50 μM) and PTZ (100 μM). Scale bars apply to all traces. **b** Statistical summary on single-channel parameters recorded in **a**. Asterisks show significance of difference from the value generated by GABA only at the corresponding patch type. *P < 0.05, **P < 0.01, ***P < 0.001, *n* = 6, Student’s paired *t* test. **c** Response amplitude histograms for receptor openings recorded in **a**. Axes legends apply to all histograms. OOP: outside-out patch, NP: nucleated patch. Note, generation of lower-conductance openings only in patches exposed to pure GABA; to improve histogram quality, recordings with GABA_A_Rs antagonists continued 3–6 times longer than that with pure GABA. **d** 5HT_3_ receptor antagonist MDL-72222 (10 μM) has no significant effect on charge transfer through s-GABA_A_Rs. Left: Example traces of whole-cell recordings. Vertical scale bar applies to both traces. Horizontal dashed lines mark the time intervals over which *I*_tonic_ was averaged for SR and SR + PTX. Right: Statistical summary. *n* = 6 and 6; Student’s unpaired *t* test

To probe single-channel GABA_A_R properties deeper, we generated all-points open-time histograms for channel openings (Fig. 2d). Histograms for recordings in the presence of GABA were best fitted with the double-Gaussian function; mode values 11.8 ± 2.22 and 33.9 ± 5.1 ms for OOPs, 13.9 ± 4.4 and 36.9 ± 7.8 ms for NPs. In the latter case, the plot clearly displayed two distinct peaks. In contrast, under all other experimental conditions, the histograms were best fitted with a single-Gaussian function. Mode values for OOPs without GABA (15.26 ± 2.71 ms), NPs without GABA (14.86 ± 2.84 ms), OOPs with SR (15.48 ± 2.92 ms) and NPs with SR (13.58 ± 2.42 ms) displayed no significant difference from the lower-mode value obtained with GABA for OOPs and NPs, respectively (*P* > 0.3, *n* = 6 and 6; Student’s *t* test for all comparisons). Our further tests proved that changes in single-channel response characteristics were not due to the side effects of GABA_A_R ligands^19,20^ (Fig. 3 and Supplementary Section 1).

### s-GABA_A_Rs modulate IPSC kinetics

**Fig. 4:**
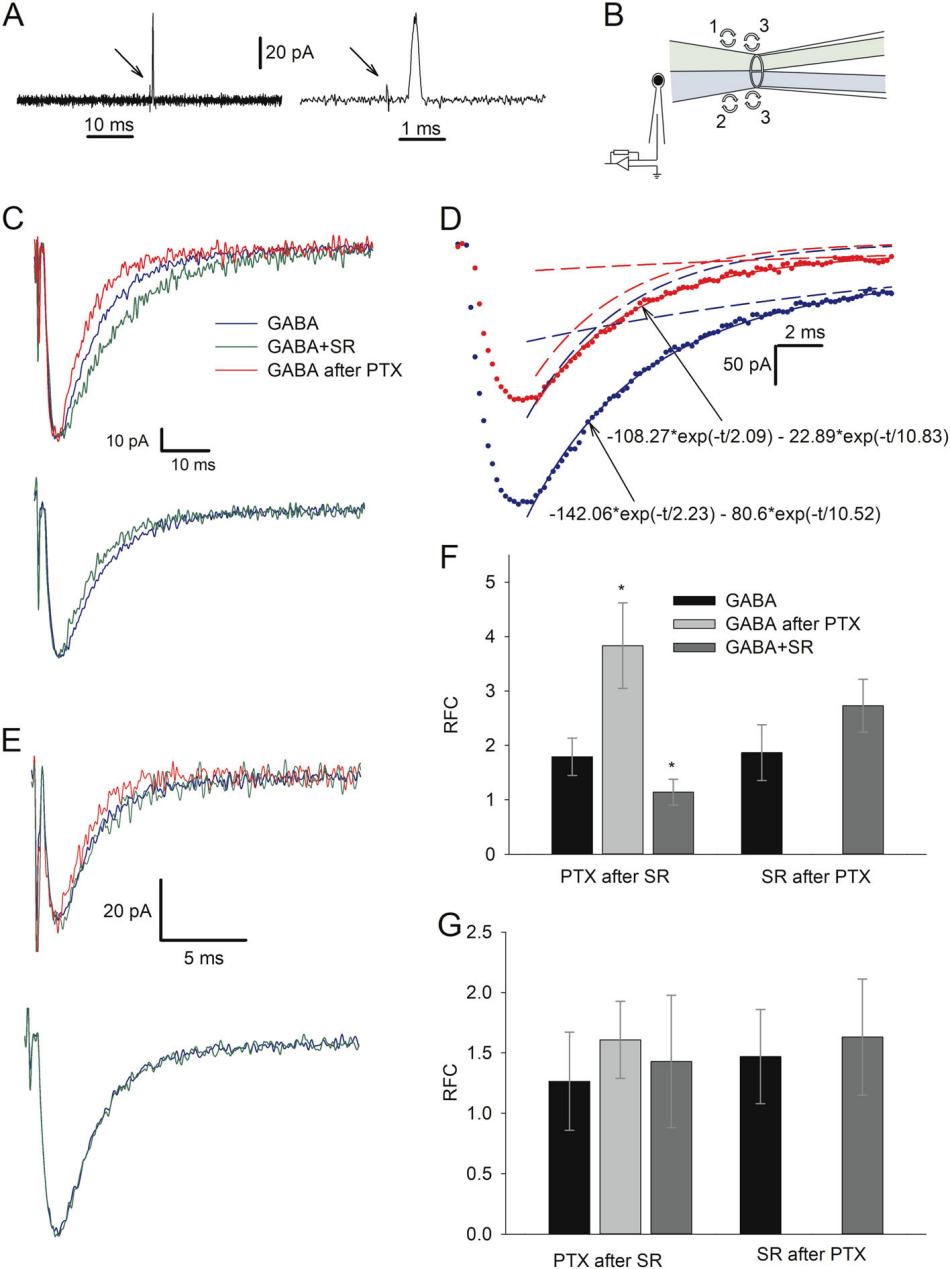
s-GABA_A_Rs modulate IPSC kinetics in nucleated patches, but not in outside-out patches. **a** Open-pipette test of solution exchange time at long (left) and short (right) time scale. Distilled water (with lower conductivity) and aCSF swap at the recording pipette tip with a time constant of 50–100 μs. Arrows point to electrical switch artefact; vertical scale bar applies to both traces. **b** Illustration of rapid solution application system (schematic) with *θ*-glass pipette, which applies two different solutions at NP placed at a patch pipette. Numbers denote sequence of drug cocktail replacements in *θ*-glass pipette channels: 1—GABA → GABA + SR; 2—aCSF with no GABA_A_R ligands → aCSF with PTX; 3—GABA + SR → GABA and aCSF with PTX → aCSF with no GABA_A_R ligands. During solution replacement time periods, the patch was exposed to the solution flowing from the “bottom” channel. **c** Inhibitory currents generated in NPs in response to short-term application of GABA_A_Rs ligands. Sequence of solution switches for top panel (three traces): no ligands—GABA (2 mM); SR (200 μM)—GABA + SR; incubation in PTX (25 μM); no ligands—GABA. For bottom panel (two traces): no ligands—GABA; incubation in PTX; SR—GABA + SR. Responses were normalized to peak amplitude of response generated by GABA in “no ligands—GABA” switch; each response is an average of 3–6. GABA_A_R ligands in applied solution are colour-coded, codes apply to **c**–**e**. **d** Analysis paradigm for response-decay kinetics with double-exponential fitting. Solid lines through data points: best-fit double-exponential functions; dashed lines of the same colour are plots of the fast and slow components alone. Blue: application of GABA; red: application of GABA after incubation in PTX. Decay components ratio of fast (*τ* = 2.23) to slow (*τ* = 10.52) component generated by GABA was obtained as 142.06/80.6 = 1.76; incubation in PTX-augmented decay components ratio to 108.27/22.89 = 4.73. **e** Same as **c**, but solutions applied at OOP. **f** Statistical summary on ratios of fitting coefficients (RFC) for recordings from NPs. Colour codes apply to **f** and **g**. **g** Statistical summary on RFC for recordings from OOPs. Asterisks denote significance of difference from ratio generated by GABA in “no ligands—GABA” switch; *P < 0.05, *n* = 6, Student’s paired *t* test

Next, we asked whether s-GABA_A_Rs can be activated by the synaptic GABA efflux. To assess this, we tested whether and to what extent does s-GABA_A_Rs shape IPSC kinetics. Our working hypothesis was that s-GABA_A_Rs are sensitive to GABA, and IPSC decay profiles should consist of two exponents, with one exponent generated predominantly by s-GABA_A_Rs and another predominantly by conventional GABA_A_Rs. To distinguish between the effects produced by the two receptor subtypes, we again used difference in action mechanisms of SR and PTX. We presumed that in the solution containing PTX, but not GABA, PTX would gradually block s-GABA_A_Rs, binding inside the ion channels when they open spontaneously, but leave intact the conventional GABA_A_Rs, since they open only after GABA binding. If after incubation in PTX, a pulse of GABA arrives, the ratio of fitting coefficients (RFC) of two IPSC decay exponents should be biased in favour of GABA-dependent GABA_A_Rs (see Fig. 4d legend for more details). In turn, the incubation in SR with subsequent GABA + SR pulse should suppress equally the effect of conventional GABA_A_Rs and s-GABA_A_Rs.

To test the hypothesis, we performed a rapid solution application experiment (Fig. 4). Since it is virtually impossible to wash out PTX from GABA_A_Rs during the characteristic lifetime of NP (3–7 min), the sequence of ligands application was first set as: GABA (2 mM)—GABA (2 mM) + SR (0.2 mM)—2–3 min incubation in PTX (50 μM)—GABA (2 mM).

Bi-exponential fitting of response-decay profiles generated in NPs by GABA after incubation in PTX displayed a higher RFC, compared with responses generated under control: 3.83 ± 0.78 vs. 1.79 ± 0.34, *P* = 0.037, *n* = 9, Student’s paired *t* test. However, the RFC of responses generated by GABA + SR unexpectedly displayed significantly lower values than that generated by the pure GABA: 1.14 ± 0.23 vs. 1.79 ± 0.34, *P* = 0.042, *n* = 9, Student’s paired *t* test (Fig. 4c–f).

Thus, we found that PTX and SR change the RFC of GABA-generated phasic response in opposite directions. But, were both effects generated by s-GABA_A_Rs? To clarify this, we again used the phenomenon of high-affinity binding of PTX to GABA_A_R. In the absence of GABA, PTX should selectively and almost irreversibly block s-GABA_A_Rs; thus, if the effect of SR does not develop after removal of PTX from the perfusion solution, this would mean that the SR-generated bias under control was due to s-GABA_A_Rs.

Therefore, we repeated the experiment with a modified sequence of the applied solutions: GABA, incubation in PTX (3 min), GABA + SR. Indeed, this prevented the development of SR-generated deceleration of decay kinetics; the RFC generated by GABA + SR did not differ significantly from that generated by pure GABA: 2.72 ± 0.49 vs. 1.86 ± 0.52, *P* = 0.22, *n* = 6, Student’s paired *t* test (Fig. 4c–f).

In contrast to NPs, pharmacological manipulations had no significant effect on RFC obtained for OOPs (Fig. 4e–g): *P* > 0.4 for all comparisons, *n* = 6–9, Student’s paired *t* test. This suggests the critical role of cytoplasmic factors in the development of GABA-dependent effects of s-GABA_A_Rs on IPSC kinetics.

GABA_A_R antagonists did not cause a significant effect on the absolute values of IPSC decay time constants and, predictably, lowered the response amplitudes (Supplementary Section 2).

**Fig. 5:**
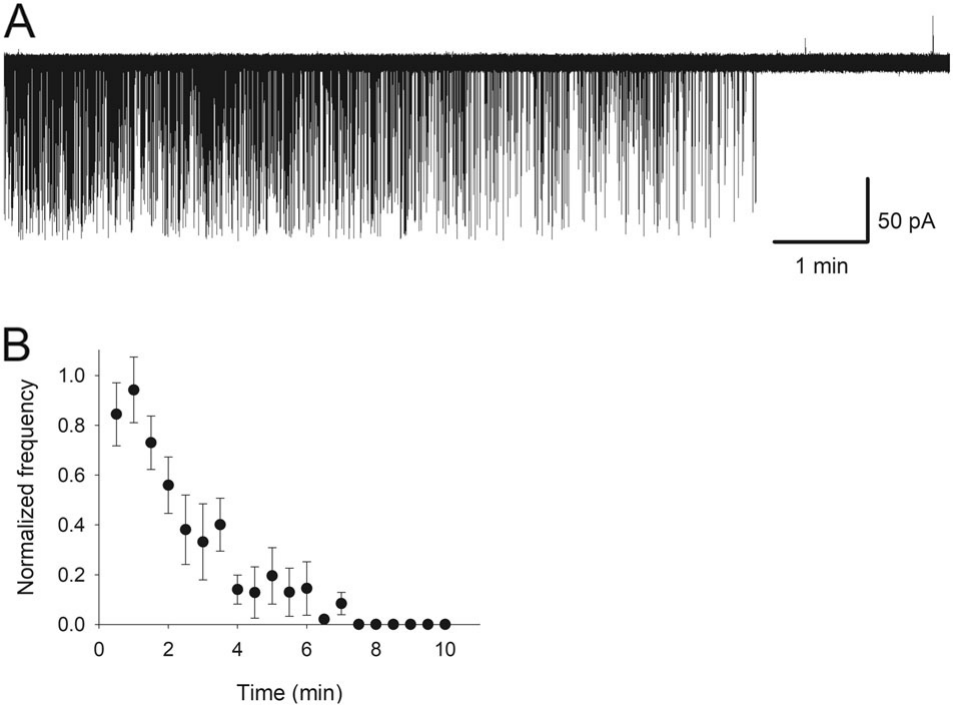
Lowered Ca^2+^ concentration combined with application of ryanodine block spontaneous GABA release. **a** Continuous recording of spontaneous IPSCs with 100 μM ryanodine and 0.2 mM Ca^2+^ in the perfusion solution. **b** IPSCs frequency normalized to control over time; *n* = 5 recordings

To further test the working hypothesis under more physiological conditions, we studied the whole-cell response kinetics in acute hippocampal tissue, where the GABA-ergic responses were induced by electrical stimulation of the perforant path. Here we combined the application of ryanodine and lowered Ca^2+^ concentration to prevent the block of GABA-dependent GABA_A_Rs due to spontaneous transmitter release during incubation in PTX (Fig. 5 and Supplementary Section 3).

**Fig. 6:**
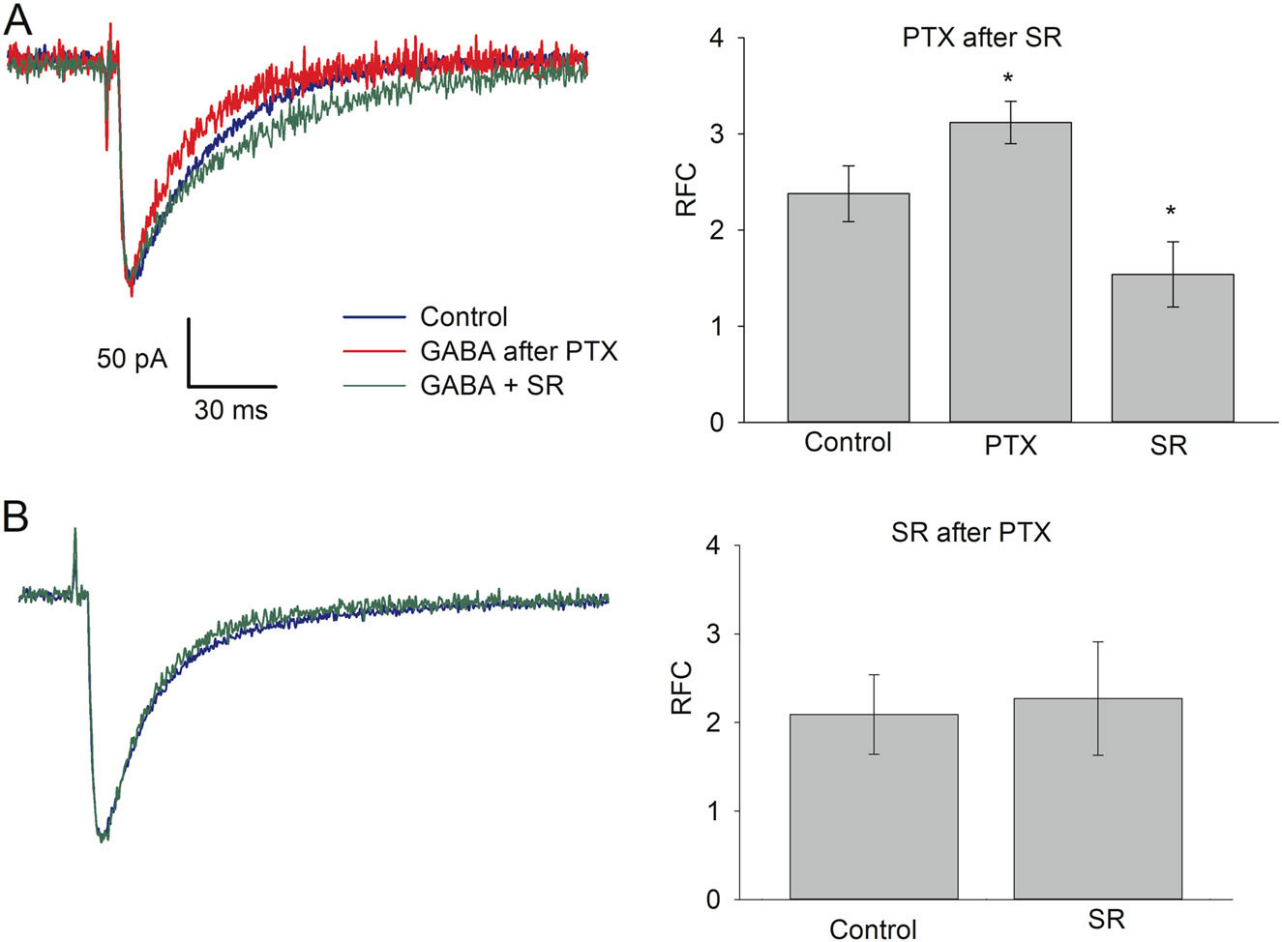
s-GABA_A_Rs modulate whole-cell response kinetics. Traces: example evoked IPSCs recorded from DGCs in acute tissue. Bar charts: statistical summary on RFC. GABA_A_R ligands in the perfusion solution are colour-coded. Sequences of ligands in the perfusion solution for **a** Control (no ligands)—SR (0.5 μM)—ryanodine (100 μM) with lowered Ca^2+^ (0.2 mM) for 10 min—ryanodine with lowered Ca^2+^ + PTX (50 μM) for 5 min—no ligands. For **b** Control—ryanodine for 10 min with lowered Ca^2+^—ryanodine with lowered Ca^2+^ + PTX for 5 min—SR. Responses were normalized to peak amplitude of response generated under control. Scale bars apply to **a**, **b**. Asterisks denote significance of difference from control, *P < 0.05, *n* = 6, Student’s paired *t* test

As expected, the application of 50 μM PTX for 5 min generated significant increase of RFC compared with control: 3.12 ± 0.22 vs. 2.38 ± 0.29, *P* = 0.038, *n* = 11, Student’s paired *t* test. In turn, SR induced significant deceleration of the IPSC kinetics: RFC 1.54 ± 0.34 vs. 2.38 ± 0.29, *P* = 0.039, *n* = 10, Student’s paired *t* test (Fig. 6). Again, pre-incubation in PTX prevented the SR effect, thus demonstrating its generation by s-GABA_A_Rs: RFC 2.27 ± 0.64 when SR was added after PTX vs. 2.09 ± 0.45 under control, *P* = 0.71, *n* = 6, Student’s paired *t* test (Fig. 6b).

### s-GABA_A_Rs regulate AP generation and signal integration properties of DGCs

**Fig. 7:**
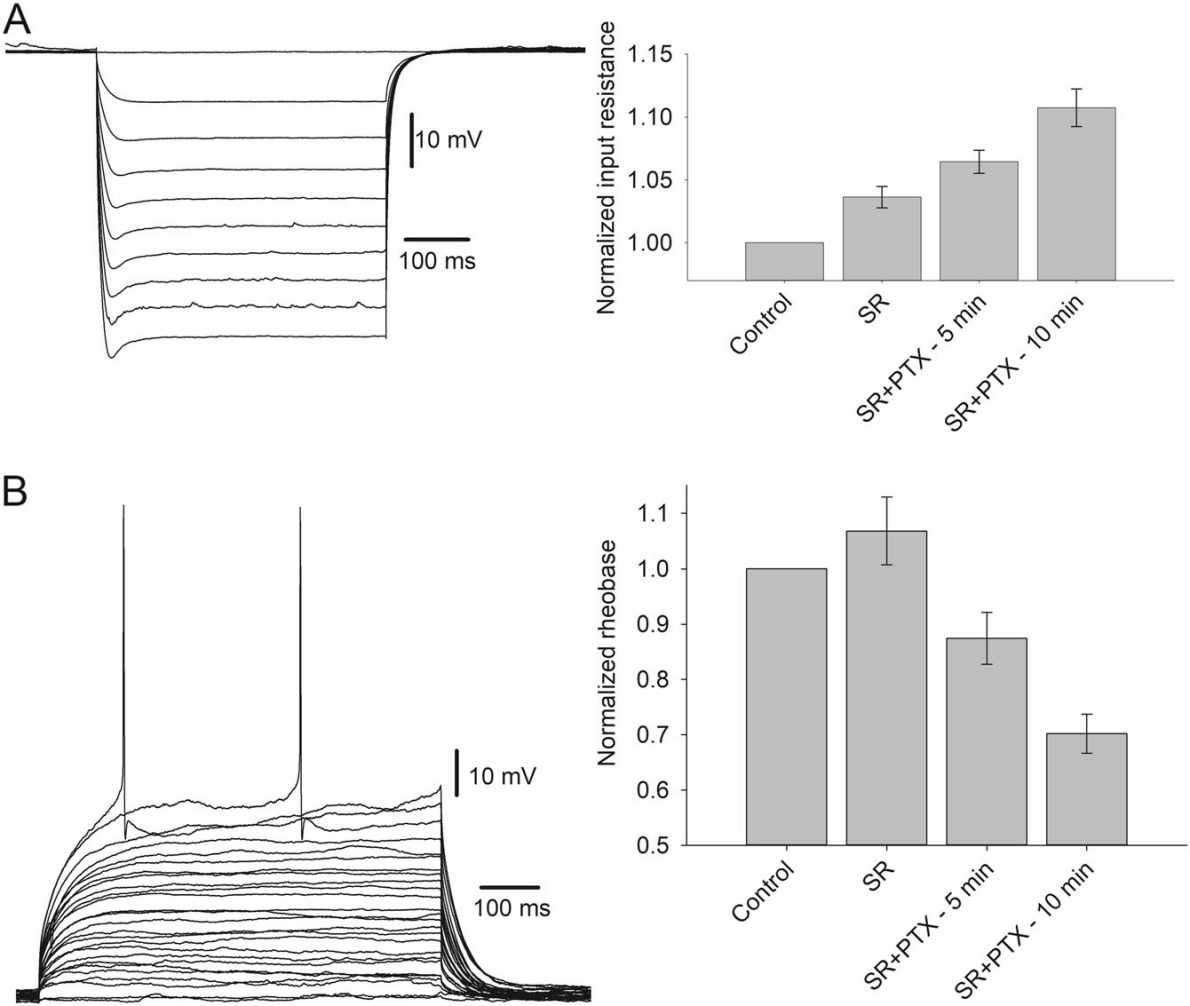
s-GABA_A_Rs control neural cell excitability. **a** Modulation of input resistance. Left: Whole-cell current clamp, example voltage responses generated by current injection from −50 to −450 pA, in 50 pA steps. Right: Statistical summary on input resistance, values normalized to control. **b** Modulation of rheobase. Left: Example recording, current injections from 5 to 125 pA in 5 pA steps. Right: Statistical summary on rheobase, values normalized to control

**Fig. 8:**
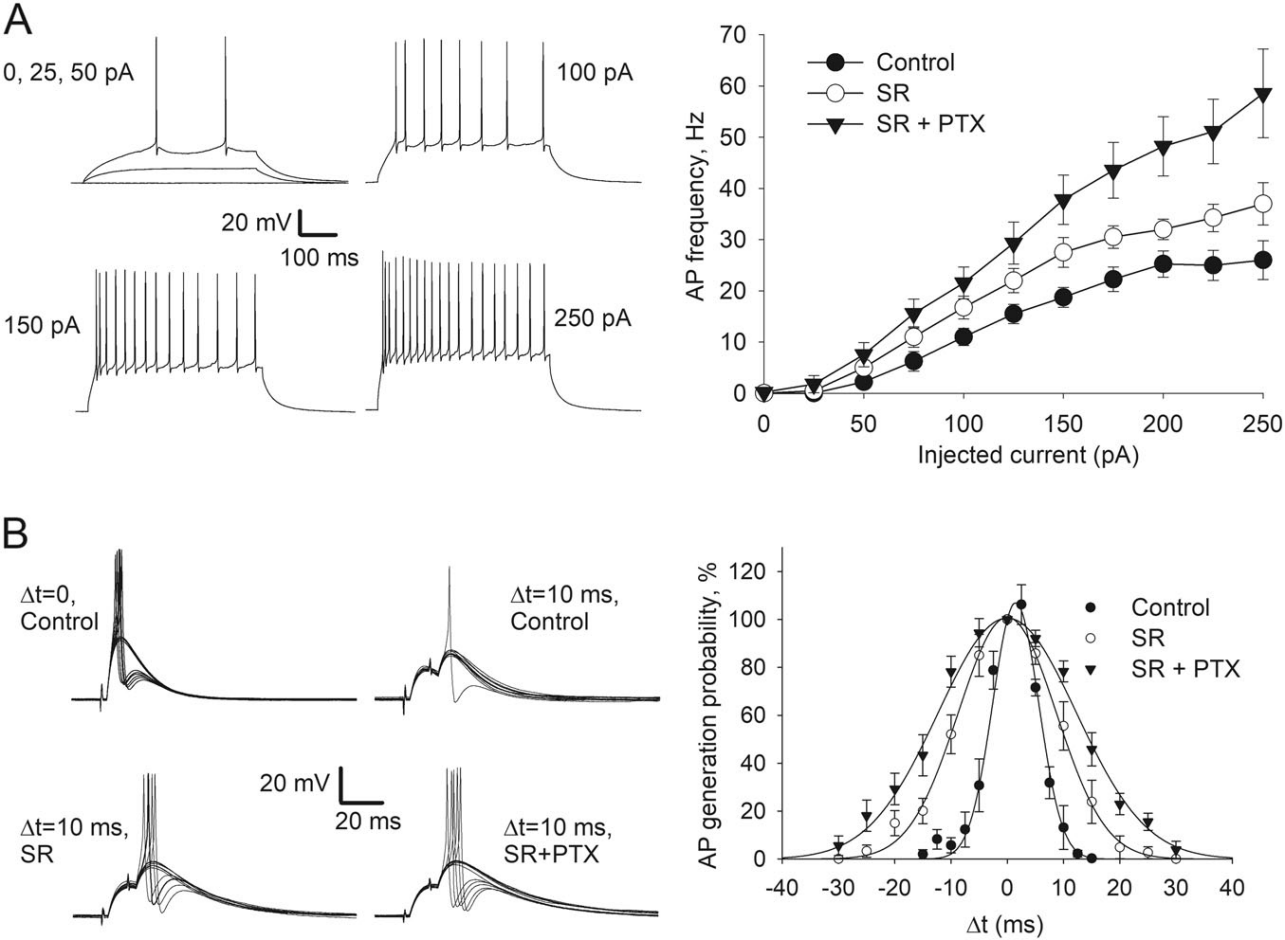
s-GABA_A_Rs modulate action potential generation and coincidence detection. **a** APs generated in response to 0.5 s current injection. Left: Example traces generated with different amperage, as indicated. Right: Plots of “amperage—AP number” dependence under control conditions and when SR (25 μM) and SR + PTX (50 μM) added to perfusion solution. **b** Coincidence detection experiment. Left: Example current traces showing recordings from DGCs upon stimulation of two perforant path pathways at inter-stimulus intervals (Δ*t*) of 0 and 10 ms under control and when SR (25 μM) or SR + PTX (50 μM) added, as indicated. Right: Histogram showing AP generation probability normalized by values obtained at Δ*t* = 0; bin width, 2.5 ms for controls, 5 ms when SR and SR + PTX added

Since s-GABA_A_Rs input in whole cell is sufficient for significant impact on response kinetics (Fig. 6) and neuronal excitability (Fig. 7 and Supplementary Section 4), we next asked whether s-GABARs modulate the action potential (AP) generation. To clarify this, we injected increasing amounts of depolarizing current and recorded the frequency of the generated APs at each current step (Fig. 8a). We found that both SR and SR + PTX significantly biased the “current–frequency” relationship. Two-way ANOVA with GABA_A_R ligands (SR 25 μM and SR 25 μM + PTX 50 μM) effect as factor 1 and injected amperage as factor 2 demonstrated highly significant impact of both factors on cell-firing frequency. For factor 1: F_(2,240)_ = 46.83, *P* = 6.73 × 10^–18^; SNK: *P* < 0.05 for all paired comparisons. For factor 2: F_(10,240)_ = 55.36, *P* = 1.06 × 10^–56^; SNK: *P* > 0.05 for data groups at 0, 25 and 50 pA. For factor 1 × factor 2: F_(20, 240)_ = 2.34, *P* = 0.0014.

Finally, to illustrate the role of s-GABA_A_Rs in signal integration, we performed a coincidence detection experiment (Fig. 8b). Here the probability of spiking under control conditions rapidly decreased when one of two stimuli was shifted in time from another with 2.5 ms steps; a Gaussian fit gave the standard deviation value as *σ* = 4.1 ± 1.3 ms (*n* = 6 cells). We then repeated the stimulation protocol (with 5 ms steps) with SR (25 μM) and SR + PTX (50 μM) in perfusion solution. The SR significantly prolonged the integration time window: *σ* = 8.7 ± 1.8 vs. *σ* = 4.1 ± 1.3 in control, *P* = 0.036, *n* = 5 and 6, Student’s *t* test. In turn, SR + PTX significantly prolonged integration window compared to SR alone: *σ* = 12.6 ± 1.7 vs. *σ* = 8.7 ± 1.8, *P* = 0.044, *n* = 6 and 5, Student’s *t* test.

## Discussion

In the present study, we performed a detailed research of the s-GABAAR-mediated tonic inhibition and revealed that these receptors provide the vast majority of tonic GABA-independent inhibitory conductance in DGCs. The functional consequence of s-GABAR conductance is to alter the basic membrane properties, shape IPSC kinetics, perturb excitability and narrow the temporal window of coincidence detection.

The significant input of s-GABA_A_Rs in inhibitory conductance of the DGCs (Fig. 1b) makes these receptors an important actor regulating signal filtering, and potentially a perspective drug target. However, was this tonic current delivered purely by s-GABA_A_Rs, but not by GABA_A_Rs, that bind GABA with a high-enough affinity to prevent it from being displaced by SR? Two observations suggest that the latter is not the case. First, in our previous study, we demonstrated that 25 μM of SR do not cause significant decrease of tonic conductance in DGCs compared to the effect of 0.5 μM^5^. Taking into account SR’s EC_50_ of ~0.15 μM^21^, the 25 μM concentration used in this study is very likely saturating all GABA-dependent GABA_A_Rs. This was further confirmed in our experiment, where the effects of SR 25 and 125 μM did not differ significantly (Fig. 3). Second, we have also shown previously that SR at the concentration 25 μM fully removes BIC effects generated at s-GABA_A_Rs^5^. Another explanation of the s-GABA_A_R current under SR could be that GABA_A_Rs of specific subtype exhibit a weak agonist response to SR. Single-channel recordings allowed us to clarify this point: application of SR together with GABA left s-GABA_A_Rs active, but did not trigger significant changes in s-GABA_A_Rs opening frequency and average open-time compared to those obtained without GABA_A_R ligands in perfusate (Fig. 2b, c). GABA concentration in this experiment (10 μM) was ~100 times higher than the native found in dentate gyrus^5^. This confirms that that 25 μM of SR are sufficient to displace GABA from GABA_A_Rs, but do not modify s-GABA_A_Rs conductance.

In this paper, we extended the previous findings on single receptor openings: s-GABA_A_Rs can be distinguished from the pool of conventional GABA_A_Rs as having shorter average open-time and lower opening probability, however, they have similar conductance (Figs. 2 and 3). The double-Gaussian distribution of single-channel open-time intervals generated by GABA in NPs (Fig. 2d) indicates the presence of two functionally distinct populations of receptors. It is worth to note that the double-Gaussian optimal fit of single-channel open-time distributions was demonstrated earlier for at least three different GABA_A_R subunit compositions^22^, with similar mode value for shorter durations, irrespective of the agonist’s type and concentration. This represents an agonist-independent input, which resembles our observations and suggests that s-GABA_A_R activity is a common element of GABA_A_R-generated inhibitory current originating from GABA_A_Rs of various subunit compositions.

s-GABA_A_Rs in OOPs have been reported previously for hippocampal primary neurons^6^, but to the best of our knowledge, not for DGCs. The presence of s-GABA_A_R openings in NPs and their absence in OOPs demonstrated in earlier work^5^ suggested critical dependence of the GABA-independent activity in DGCs on cytoplasmic factors. However, here we successfully recorded s-GABA_A_R's spontaneous openings in OOPs using low-impedance patch pipettes; thus, the inability to register them in earlier studies was probably due to s-GABA_A_R's low density on cell soma, aggravated by the tendency of GABA_A_Rs to cluster^23^.

When GABA_A_Rs are activated by GABA pulse, the block of s-GABA_A_Rs with PTX suppressed the slow component of the decay profile more than the fast one (Fig. 4), and the block of GABA binding with SR suppressed the fast component more than the slow one. This effect of SR, however, did not develop when s-GABA_A_Rs were deactivated by pre-incubation in PTX (Figs. 4 and 6), thus proving the SR-induced suppression of the fast component to be generated by s-GABA_A_Rs. On top of a general proof of ability of s-GABA_A_Rs to be activated by GABA, this observation suggests that, when activated by GABA, s-GABA_A_Rs have different single-channel response characteristics compared to their spontaneous activity state, and/or have higher affinity to GABA than the conventional GABA_A_Rs.

Studies on network signalling in the hippocampus have shown DGCs to be linear integrators of synaptic inputs coming from the entorhinal cortex^24^. To match this role, DGCs should act as strong EPSP attenuators with a high AP threshold^24,25^. Therefore, inhibition delivered by s-GABA_A_Rs, which narrows an integration window for excitatory inputs (Fig. 8b), is a key mechanism that ensures implementation of the main functional role of DGCs.

Subunit selective GABA_A_R ligands are widely recognized to be a perspective class of compounds for exploring and validating potential anti-epileptic drugs allowing fine-tuning of TLE therapy^26,27^. Therefore, an important question to be addressed in future studies is, which GABA_A_R subunit(s) turns conventional GABA_A_Rs to s-GABA_A_Rs? It has been demonstrated that scarcity of α1 subunit is correlated with resistance to anti-epileptic drugs^28^, whereas increased α1-GABA_A_R expression in hippocampus suppresses TLE development^8^. α1 subunit when incorporated into GABA_A_Rs has been shown to modulate spontaneous GABA_A_Rs gating^29^, thus making α1-GABA_A_R a good candidate for generation of at least a part of s-GABA_A_Rs effects. Positive allosteric GABA_A_R modulator zolpidem selectively increases the activity of α1-subunit-containing GABA_A_Rs partially in GABA-independent manner^30^. Apart of α1-GABA_A_R, another possible candidate to be a part of s-GABA_A_Rs functional group is α5-containing GABA_A_R: it was shown to generate SR-insensitive component of phasic current^31^ and contribute to tonic conductance^32^. Compounds such as zolpidem and propofol, which upregulate s-GABA_A_Rs activity in GABA-independent manner^6,30^, maybe thus be useful candidates to counteract TLE development.

It has long been established that amount of Cl^−^ current transfer through GABA_A_Rs has a bell-shaped dependence on temperature in a physiological temperature range^33,34^. This is specifically important for DGCs, where rise of temperature from 22–23 to 34–36 °C generates approximately twofold increase of tonic current^35^. On top of that, increase of temperature augments zolpidem-modulated inhibitory current through α1-GABA_A_Rs to a higher extent than the inhibitory current through zolpidem-insensitive GABA_A_Rs^36^. This may suggest that s-GABA_A_Rs of DGCs (selectively modulated by zolpidem) are one of the main factors implementing temperature-dependent control over inhibitory current.

Abnormal increase of intracellular Cl^−^ concentration was found earlier to slow down GABA_A_R IPSC kinetics, in particular, in IPSCs generated by α1-containing GABA_A_Rs, and to modulate neuron-firing probability^37^. This raises a question whether and to what extent s-GABA_A_Rs effects observed in our work were dependent from Cl^−^ concentration. Indeed, IPSC deceleration generated by s-GABA_A_Rs in our voltage-clamp recordings was observed under 128.5 mM intracellular Cl^−^ (see Methods), which is much higher than physiological concentration and implies existence of Cl^−^-generated bias. However, in current-clamp experiments, s-GABA_A_Rs caused a significant impact on neuronal firing under 4 mM intracellular Cl^−^, which is close to the lower limit of physiological concentrations typically found in neurons (~5–10 mM^37^). Therefore, s-GABA_A_Rs effects in our preparation cannot be reduced to impact intracellular Cl^−^ concentration on inhibitory transmission.

Both α1- and α5-GABA_A_R are modulated by a number of cytoplasmic proteins^38–40^, which are controlled by G protein-delivered signalling triggered by glutamate and GABA_B_ receptors^41–43^. Therefore, permanent block of GABA_B_ and glutamate receptors in our study may exert a stable background effect, which modifies quantitative outcome of s-GABA_A_Rs activation. This points to cytoplasmic actors as to one of the further research directions that allows comprehension of s-GABA_A_Rs functioning.

## Methods and materials

### Hippocampal slice preparation

Transverse hippocampal slices (350–400 μM thick) were used for in vitro electrophysiological recordings. Slices were prepared from 3- to 5-week-old female Sprague Dawley rats. Animals were killed by cervical dislocation after being anesthetized by an overdose of isoflurane, according to the United Kingdom Animals (Scientific Procedures) Act of 1986. After decapitation, brains were rapidly removed and dissected, and whole-brain sagittal slices were prepared with a Leica VT1200S vibratome in semi-frozen sucrose-based solution containing the following (in mM): 70 sucrose, 80 NaCl, 2.5 KCl, 4 MgCl_2_, 0.5 CaCl_2_, 15 NaHCO_3_, 10 HEPES, 1.25 NaH_2_PO_4_ and 22 glucose, equilibrated with 95% O_2_ plus 5% CO_2_, pH 7.35, 315–330 mOsm. Slices were placed in continuously oxygenated sucrose-aCSF at 35 °C for 20 min and then allowed to recover for a further 30 min at room temperature before recording. Individual slices were then transferred into recording chamber and perfused with standard ACSF containing (in mM): 119 NaCl, 2.5 KCl, 1 MgCl_2_, 2.5 CaCl_2_, 10 HEPES, 15 NaHCO_3_, 1.25 NaH_2_PO_4_ and 22 glucose, and was continuously gassed with 95%O_2_ and 5%CO_2_, pH 7.35; 290–298 mOsm. aCSF temperature during experiments was held at 33 °C, under the control of an inline heater.

### Electrophysiology

#### Whole-cell recordings

Visualized patch-clamp recordings from mature dentate granule cells were performed using an infra-red differential interference contrast imaging system. Tonic GABA_A_R-mediated currents were measured in voltage-clamp mode at holding potential *V*_hold_ = −70 mV at 33 °C in the presence of ionotropic glutamate receptor blockers, DL-APV (50 μM) and NBQX (20 μM), metabotropic glutamate receptors blocker, (S)-α-methyl-4-carboxyphenylglycine (MCPG; 250 μM) or *N*-tricyclo-[3.3.1.13,7]-dec-1-yl-2-quinoxalinecarboxamide (NPS; 10 μM) plus (RS)-α-Cyclopropyl-4-phosphonophenylglycine (CPPG; 5 μM), glycine receptor blocker, strychnine (1 μM) and GABA_B_ receptor blocker (2S)-3-[[(1S)-1-(3,4-dichlorophenyl)ethyl]amino-2-hydroxypropyl](phenylmethyl) phosphinic acid (CGP55845; 1 μM). The intracellular pipette solution for voltage-clamp experiments contained the following (mM): 120.5 CsCl, 10 KOH-HEPES, 2 EGTA, 8 NaCl, 5 QX-314 Br^−^ salt, 2 Mg-ATP and 0.3 Na-GTP; for current-clamp experiments, it contained 126 K-gluconate, 4 NaCl, 5 HEPES, 15 glucose, 1 MgSO_4_·7H_2_O, 2 BAPTA and 3 Mg-ATP; pH adjusted to 7.2 and osmolarity adjusted to 295 mOsm. Competitive antagonist SR-95531 (SR) and channel blocker picrotoxin (PTX) were used to apply partial and full block of GABA_A_Rs activity, respectively. Recordings were performed at 32–34 °C; the patch pipette resistance was 2–4 MΩ for whole-cell recordings and 3–7 MΩ for recordings from outside-out and nucleated patches. Series resistance was monitored throughout the experiments using a +5 mV step command; cells with unstable series resistance (above 25 MΩ) or unstable holding current were rejected. To stimulate the hippocampal neural tissue, electrical stimuli were delivered through bipolar-stimulating electrode placed in perforant path. Constant voltage DS2A stimulus isolators (Digitimer LTD) were used to deliver electrical stimuli to neural tissue during whole-cell recordings and activate piezoelectric actuator in rapid solution application experiments on membrane patches.

#### Outside-out and nucleated patch recordings

Outside-out patches (OOPs) and cell membrane bags containing intact nucleus and cytoplasm (nucleated patches, NPs) were pulled from dentate gyrus granule cells, and recordings were performed in voltage-clamp mode (*V*_hold_ −70 mV). Rapid solution exchange experiments were performed as described in our earlier published protocol^44^. Briefly, we used a *θ*-glass application pipette, with ~200 μm tip diameter attached to the micromanipulator. The position of the pipette was controlled by piezoelectric element, the switch time constant was 50–100 μs. One pipette channel was filled with the bath aCSF solution and another channel had GABA or GABA plus antagonist (SR or PTX). Pressure was regulated by a PDES-02DX pneumatic micro ejector (npi), using compressed nitrogen separately in each of the two channels. Solutions with GABA, GABA + SR and GABA + PTX were exchanged in a pipette channel in 7–12 s^44^; after replacement of solutions, the following series of rapid applications of one type of solution takes about 15–30 s. Therefore, since GABA_A_Rs response recovery after PTX application requires a washout for the time period from 10–15 min^45^ to several tens of minutes^46,47^, we presumed that at least a large part of s-GABA_A_Rs remains blocked by PTX during the <1 min full experimental cycle.

To prevent contamination with the spilled over GABA released from neural tissue, continuously recorded membrane patches were placed in a solution current flowing from the motionless *θ*-glass pipette, where stable concentrations of GABA_A_R ligands were maintained.

There are several technical problems that affect data interpretation in this type of experiments. First, due to the large difference in surface area between the membrane patches (especially between NPs), the number of individual ion channels, in particular, patches may vary up to an order of magnitude, with corresponding effect on the response amplitude. Another technical complication of the rapid solution exchange experiment on NPs is the turbulence in applied liquid at the side of NP opposite to the solution application side that could have a variable effect on the kinetics of the recorded responses (refer to Supplementary Section 2 for numerical data). These factors introduce a large variability to experimental readouts obtained in different recordings and may make the data hardly interpretable. To cope with that, we applied control and experimental sets of chemicals on the same patch and then used the ratios of fitting coefficients for components with fast and slow decay time constants (*τ*) rather than absolute values of amplitudes and/or *τ*.

### Acquisition and analysis

Recordings were obtained using a Multi-Clamp 700B amplifier (Molecular Devices), filtered at 4–8 kHz, digitized at 10 kHz and stored on a PC. pClamp/Clampfit 10 × software (Molecular Devices) was used for data storage and off-line analysis. For nonlinear fitting of single- and double-Gaussian and double-exponential functions, we used Wolfram Mathematica 10 software package.

#### Analysis of tonic currents

For analysis of tonic whole-cell currents, mean values of holding current were averaged at 30 s intervals. The shift of the tonic current was calculated as the difference between the holding current values (Δ*I*_hold_) measured at stable baseline intervals before and after the application of an antagonist/channel blocker. The tonic s-GABAAR-mediated current was measured as the outward shift in holding current following application of PTX (50 μM) in the continued presence of SR (25 μM).

Changes in root mean square (RMS) noise have also been used to reflect changes in tonic GABA_A_R-mediated conductance and have been used because they are unaffected by current drift. Although RMS noise decreased in experiments in which tonic currents were blocked, this measurement is confounded by the presence of synaptic currents. Moreover, RMS noise is nonlinearly related to current and can paradoxically decrease when tonic currents increase^48^. We, therefore, only used RMS noise as a measure in experiment in which we were trying to block the tonic current. The values of the RMS noise were calculated for 200 ms epochs free of synaptic events. The change of RMS noise (ΔRMS) was calculated as the difference between the values before and after the application of antagonist(s). We obtained RMS noise values with calculation algorithms built-in into Clampfit 10 software.

Inhibitory charge transfer with phasic events and through tonically open s-GABA_A_Rs was obtained as *Q*_ph_ = *F* × *Q*_AV-ph_ × Δ*t* and *Q*_t_ = Δ*I*_hold_ × Δ*t*, respectively. Here *Q*_ph_—overall phasic charge transfer, *F*—frequency of phasic events; *Q*_AV-ph_—average charge transferred with individual phasic event (area under event’s curve); *Q*_t_—overall tonic charge transfer, Δ*I*_hold_—difference between holding current under SR and SR + PTX; Δ*t*—time interval over which charge transfer was calculated; see Fig. 1a for graphical representation.

Synaptic events were automatically detected and stored with Clampfit 10 detection algorithm. For spontaneous IPSCs area under curve was calculated for the space between the event peak and baseline obtained as average current for 3 ms before the IPSC occurrence. For tonic current, the area under the curve was calculated by the Δ*I*_hold_ multiplied by the time (60 s).

#### Analysis of the single-channel recordings

Application of GABA at outside-out patches (OOPs) and nucleated patches (NPs) evoked single-channel openings to two conductance levels: 37.2 ± 6.4 pS and 17 ± 9.3 pS (see Figs. 2a and 3a). The larger conductance level contributed 92.8% of the single-channel current, whereas subconductance level contributed 7.2% of the current; thus, the larger main conductance level was used when (possible) changes of single-channel conductance were compared under different experimental conditions.

The opening frequency of GABA_A_R channels was calculated as *N*/Δ*t*, where *N* is the number of openings and Δ*t* is the time of recording. *N* was counted using a detection threshold of 1.6 pA more negative than the mean baseline and a minimum opening time of 0.4 ms.

It was virtually impossible to determine accurately the number of channels in a membrane patch; however, in our preparation, the vast majority of channel openings were single-level events. In a case where there were multiple levels of channel openings, only the level with the highest conductance was analysed. This prevented us from overestimating opening-frequency increase, since in multi-channel patch, increased opening frequency would be accompanied by increase of proportion of multi-level events. Values for the average open-time of single channel were obtained with threshold-detection algorithm of Clampfit 10 × software.

To calculate and visualize the average open-time characteristic for different receptor subtypes, we constructed all-points histograms and fitted them with a double-Gaussian function:

$$F = \frac{{p_1e^{ - \frac{{\left( {n - m_1} \right)^2}}{{2\sigma _1^2}}}}}{{\sigma _1\sqrt {2\pi } }} + \frac{{p_2e^{ - \frac{{\left( {n - m_2} \right)^2}}{{2\sigma _2^2}}}}}{{\sigma _2\sqrt {2\pi } }}$$

where *m*_1_ and *m*_2_ are the mode values of Gaussians, *σ*_1_ and *σ*_2_ are the standard deviations of corresponding modes, *n* is the value of electrical current and *p*_1_ and *p*_2_ are the fitting constants. The general algorithm of multi-Gaussian histogram construction, fitting and interpretation were adapted from the works of Bennett and Kearns^49^, and Traynelis and Jaramillo^48^.

#### Analysis of phasic responses

Decay profiles of phasic responses recorded from NPs and whole-cell were fitted with double-exponential function

$$\Delta I = - a_1e^{ - \frac{t}{{\tau _1}}} - a_2e^{ - \frac{t}{{\tau _2}}}$$

where Δ*I* is a difference between the current recorded at baseline and at time *t*, *e*—the Euler’s constant, *a*_1_ and *a*_2_—fitting constants and *τ*_*1*_ and *τ*_*2*_—decay time constants.

#### Data analysis in step current injection experiments

DGC input resistance was calculated as the gradient of the straight line best fitting the VI plot of the anti-peak voltage amplitude against the corresponding hyperpolarising current of 500 ms duration from −50 to −450 pA.

The value of DGC rheobase was measured in the experiment, where 1 s current injections with +5 pA incrementing steps were delivered until propagation of the first action potential.

Frequency of action potential generation (Hz) was measured at 0.5 s time intervals, where depolarizing current of 25–250 pA was injected in 25 pA steps.

#### Data analysis in coincidence detection experiment

Here, two stimulation electrodes were placed in the perforant path area, and the stimulation intensity was set so that when the two pathways were stimulated simultaneously, the patched DGC generated AP in ~50% of the trials (threshold stimulation). After application of SR and SR + PTX, stimulation intensity at both electrodes was re-adjusted to match the spiking probability observed under the control. We then applied a single-Gaussian nonlinear fitting (similar approach as to open-time data from single-channel recordings) and used the changes of the standard deviation value (*σ*) for quantification of SR and SR + PTX effect on coincidence detection window.

GABA receptor antagonists, MCPG, CPPG, NPS, NBQX, CGP55845 and MDL-72222, were purchased from Tocris Bioscience. All other chemicals were purchased form Sigma-Aldrich. All data are given as mean ± standard error of mean. For statistical calculations, Student’s paired *t* test, Student’s unpaired *t* test, one-way and two-way analysis of variance (ANOVA) with Student–Newman–Keuls post hoc test were applied as indicated.

## Electronic supplementary material

Supplementary materials
