# Supplementary material for: Spontaneously opening GABAA receptors play a significant role in neuronal signal filtering and integration
Source: Cell Death Dis. 2018 Jul 24;9(8):813. doi: 10.1038/s41419-018-0856-7 (PMC6057890; doi:10.1038/s41419-018-0856-7)
Supplement: Supplementary file 1 — Supplementary materials [file 41419_2018_856_MOESM1_ESM.docx]

**Supplementary materials**

**Supplementary section 1. s-GABA_A_Rs-attributed conductance is not due to an off-target effects of GABA_A_R ligands**

Recordings from membrane patches demonstrated clearly GABA-independent s-GABA_A_Rs single-channel openings (Fig. 2). However, were these openings indeed generated by s-GABA_A_Rs, or propagated due to side effects of the compounds used for their isolation? To clarify this, we repeated the experimental protocol for single-channel recordings, but with higher concentration of SR (125 μM) and with alternative GABA_A_R ligands with action mechanisms similar to those of SR and PTX: antagonist with competitive action mechanism BIC (50 μM), and open channel blocker pentylenetetrazole (PTZ, 100 μM), respectively (Fig. 3A).

125 μM SR and 50 μM BIC, being applied at OOPs and NPs, did not generate significantly different effects on channel conductance, opening frequency and average open time, compared to those of SR 25 μM (Fig. 3B): P>0.25 for all comparisons, n=6, 6, Student’s t-test. This confirmed first that 25 μM concentration of SR fully antagonises GABA binding and, second, suggests that spontaneous openings do not arise due to a side effect of SR. In addition, applying PTZ instead of PTX, we obtained an identical suppression of single-channel openings (Fig. 3A), thus arguing against such a silencing to be generated by an off target effect of PTX.

To study (possible) effects of GABA_A_R ligands on receptor conductance, we constructed all-point amplitude distributions of single-channel openings (Fig. 3C). When GABA was applied, histograms clearly displayed two peaks corresponding to larger and smaller conductance level, and were best fitted with double-Gaussian function. Mode values for smaller amplitudes (subconductance level) were fitted as 1.24±0.22 pA for NPs, 1.21±0.18 pA for OOPs; for larger amplitudes (main conductance level) as 2.65±0.13 pA for NPs, 2.67±0.11 pA for OOPs. Application of SR and BIC abolished the lower amplitude events indicating that these were GABA-generated, rather than spontaneous in origin, thus giving one more functional property which allows separation of s-GABA_A_Rs and conventional GABA_A_Rs effects.

Single-Gaussian fitting generated mode amplitude values for SR 25μM 2.59±0.14 pA in NPs, 2.66±0.27 pA in OOPs; for SR 125 μM 2.63±0.15 pA in NPs, 2.7±0.23 pA in OOPs; for BIC 50 μM 2.61±0.19 pA in NPs, 2.69±0.24 pA in OOPs (Fig. 3C). All mode amplitude values obtained under GABA_A_R antagonists displayed no significant difference from larger amplitude values generated by GABA only in NPs and OOPs, respectively: P>0.4, n=6, 6 for all comparisons, Student’s t-test.

We performed two additional experiments to control for potential off-target effects mimicking s-GABA_A_Rs activity. First, we applied PTZ (100 μM), which caused a complete suppression of single channel-openings, similarly to PTX (Fig. 3A). Second, we assessed if the change in whole cell holding current could be due to an effect on 5-HT_3A_ receptors^49,50^, using the 5-HT_3A_-selective inverse agonist MDL-72222 (Fig. 3D). We found no significant effect of MDL-72222 (10 μM) on holding current (ΔI_hold_ = -0.19±1.15 pA, P=0.88, n=6, paired Student’s t-test) or the tonic current suppressed by PTX: 13.05±0.97 pA in control vs. 10.83±1.55 pA under MDL-72222, P=0.23, n=6, 6, Student’s t-test.

**Supplementary section 2. Effects of pharmacological interventions on IPSC amplitudes and decay time constants in rapid solution application experiment.** In contrast to RFCs, in this experiment absolute values of decay time constants (τ) did not display significant modifications associated with pharmacological interventions.

In NPs, τ of fast decay component generated by pure GABA was 2.96±1.13 ms, τ of slow decay component was 13.44±6.86 ms. Application of GABA+SR after GABA generated τ values of 3.18±1.09 ms and 14.77±5.29 ms; application of GABA+SR after incubation in PTX generated τ values of 2.69±0.97 and 12.34±3.58 ms; application of GABA after PTX generated τ values of 2.53±0.89 and 10.08±4.33 ms for fast and slow component, respectively; P>0.4 for all comparisons to GABA-generated τ in intact patch, n=6-9, Student’s paired t-test.

τ values generated in OOPs were as follows. Pure GABA, intact patch: 0.97±0.21 for fast component, 3.16±1.24 for slow component; GABA+SR after GABA: 1.02±0.18 and 2.88±0.91; GABA+SR after PTX: 1.11±0.26 and 3.06±1.23; GABA after PTX: 0.94±0.19 and 2.54±1.1. Again, τ values for fast and slow components generated by GABA+SR and GABA after PTX did not differ significantly from those generated by GABA only in intact patch: P>0.5 for all comparisons, n=6, Student’s paired t-test.

Amplitude of responses generated in NPs by pure GABA was 72.2±18.3 pA; GABA+SR after pure GABA reduced response amplitude to 27.3±14.6 pA, GABA+SR after incubation in PTX generated 22.57±10.91 pA amplitude, GABA after incubation in PTX generated 24.7±11.8 pA amplitude. Response amplitude generated by pure GABA in intact NPs, as expected, was significantly higher than in all other cases: P<0.05 for all comparisons, n=6-9, Student’s paired t-test.

Amplitude of responses generated in OOPs were as follows. Pure GABA, intact patch: 44.23±12.27 pA; GABA+SR after GABA: 15.22±7.41 pA; GABA+SR after incubation in PTX: 12.46±5.14 pA; GABA after incubation in PTX: 17.06±7.28 pA. As in NPs, amplitude generated by pure GABA in intact OOPs was significantly higher than in all other cases: P<0.05 for all comparisons, n=6, Student’s paired t-test.

**Supplementary section 3. Spontaneous release of GABA in acute tissue can be prevented by ryanodine and lowered Ca^2+^ concentration.** To prevent the block of GABA-dependent GABA_A_Rs due to spontaneous transmitter release during incubation in PTX, we lowered the concentration of Ca^2+^ in the perfusion solution to 0.2 mM and applied 100 μM ryanodine 10 minutes prior to PTX. Our control experiment proved that such an approach suppresses spontaneous synaptic activity (Fig. 5).

**Supplementary Section 4.** **s-GABA_A_Rs modulate excitability of DGCs**

Here we asked whether s-GABA_A_Rs input in whole cell effects is sufficient for significant impact on neuronal excitability. We performed step current injections in the current-clamp mode, which allowed the calculation of cell input resistance and rheobase (Fig. 7). When -50 pA hyperpolarising current injections were applied with 25 μM SR in the perfusion solution, the input resistance was obtained as 167.2±6.7 MΩ, with a small increase from control value of 161.5±6.8 MΩ. Subsequent addition of PTX 50 μM with SR caused a progressive increase in input resistance, demonstrating the use-dependent nature of PTX effect: 5 minutes of PTX exposure increased the input resistance value to 171.9±7.6 MΩ; after 10 minutes, PTX increased input resistance to 179.02±9.1 MΩ. If we compared raw input resistance using a one-way ANOVA, no significant influence of GABA_A_R ligands was found, which reflects a large inter-cell variability: F_(3,28)_=1.19, P=0.33. In order to account for inter-cell variability, we normalized input resistance in SR and SR+PTX to control values obtained in the same cell (Fig. 7A). After such a transformation one-way ANOVA demonstrated high significance of ligands effect: F_(3,28)_=21.12, P=2.36×10^-7^; SNK: P<0.05 for all compared pairs.

Next, we assessed how s-GABA_A_Rs influence the cell rheobase (Fig. 7B). We applied 1 second long 5 pA depolarising current steps to obtain a rheobase value, and found that addition of 25 μM SR changed rheobase to 85±9.84 pA from 74.29±12.25 pA under control. Subsequent application of 50 μM PTX caused a progressive rheobase value reduction: to 72.5±9.93 pA after 5 minutes of incubation, and to 60.63±8.2 after 10 minutes of incubation. Again, one-way ANOVA on raw data did not confirm significant effect of SR and SR+PTX: F_(3,28)_=0.98, P=0.42. However, being applied on data normalized to control, one-way ANOVA demonstrated a significant effect of SR and SR+PTX: F_(3,28)_=3.34, P=0.033; SNK: P<0.05 for Control vs. SR+PTX for 5 minutes and Control vs. SR+PTX for 10 minutes.
